# Ibrutinib as a potential therapeutic for cocaine use disorder

**DOI:** 10.1038/s41398-021-01737-5

**Published:** 2021-12-08

**Authors:** Spencer B. Huggett, Jeffrey S. Hatfield, Joshua D. Walters, John E. McGeary, Justine W. Welsh, Trudy F. C. Mackay, Robert R. H. Anholt, Rohan H. C. Palmer

**Affiliations:** 1 Behavioral Genetics of Addiction Laboratory, Department of Psychology at Emory University, Atlanta, GA USA; 2 Department of Genetics and Biochemistry and Center for Human Genetics, Clemson University, Greenwood, SC USA; 3 Department of Psychiatry and Human Behavior, Brown University, Providence, RI USA; 4 Providence Veterans Affairs Medical Center, Providence, RI USA; 5 Department of Psychiatry and Behavioral Sciences, Emory University School of Medicine, Atlanta, GA USA

**Keywords:** Pharmacogenetics, Molecular neuroscience, Pharmacogenomics, Addiction, Scientific community

## Abstract

Cocaine use presents a worldwide public health problem with high socioeconomic cost. No current pharmacologic treatments are available for cocaine use disorder (CUD) or cocaine toxicity. To explore pharmaceutical treatments for tthis disorder and its sequelae we analyzed gene expression data from post-mortem brain tissue of individuals with CUD who died from cocaine-related causes with matched cocaine-free controls (*n* = 71, *M*_age_ = 39.9, 100% male, 49% with CUD, 3 samples/brain regions). To match molecular signatures from brain pathology with potential therapeutics, we leveraged the L1000 database honing in on neuronal mRNA profiles of 825 repurposable compounds (e.g., FDA approved). We identified 16 compounds that were negatively associated with CUD gene expression patterns across all brain regions (*p*_adj_ < 0.05), all of which outperformed current targets undergoing clinical trials for CUD (all *p*_adj_ > 0.05). An additional 43 compounds were positively associated with CUD expression. We performed an *in silico* follow-up potential therapeutics using independent transcriptome-wide in vitro (neuronal cocaine exposure; n = 18) and in vivo (mouse cocaine self-administration; *n* = 12–15) datasets to prioritize candidates for experimental validation. Among these medications, ibrutinib was consistently linked with the molecular profiles of both neuronal cocaine exposure and mouse cocaine self-administration. We assessed the therapeutic efficacy of ibrutinib using the *Drosophila melanogaster* model. Ibrutinib reduced cocaine-induced startle response and cocaine-induced seizures (*n* = 61–142 per group; sex: 51% female), despite increasing cocaine consumption. Our results suggest that ibrutinib could be used for the treatment of cocaine use disorder.

## Introduction

To date, there are no medications approved by the Food and Drug Administration (FDA) for human CUD or cocaine toxicity. Approximately 18.1 million people use cocaine globally each year [1] and roughly 30% of those users reside in the United States [2] (US). In the US, nearly 1 million individuals meet criteria for cocaine use disorder [2] (CUD) and 13% of the individuals in North American substance use treatment facilities are treated for CUD [1]. Cocaine use increases risk for cardiovascular disease, seizures, and mental health disorders and contributes to roughly 40% of drug-related emergency room visits in the US [3]. Cocaine exerts adverse neurological effects in the brain, and at high doses can lead to cardiovascular disease and death. Since 1999, cocaine-involved overdose deaths have increased by 416% in the US and resulted in 15,883 deaths in 2019 [4].

While behavioral treatments for CUD exist, they have limited efficacy and are plagued by high dropout rates [5]. Over 20 pharmcologic agents are currently being tested for clinical trials for CUD. Most of these medications include antidepressants, antipsychotics, psychostimulants, cognitive enhancing drugs, anxiolytics, repurposed medications for other substance use disorders, anticonvulsants/muscle relaxants, and dopamine agonists [6]. These treatments generally have mixed efficacy and often only demonstrate effects in particular subpopulations.

Medications for cocaine toxicity are also limited. Emergency room practitioners rely on beta-blockers for cocaine-induced cardiotoxicity [7] and benzodiazapines for cocaine-induced seizures [8]. Many candidate compounds for treating CUD and cocaine toxicity rely on repurposing existing medications, leveraging knowledge of cocaine’s pharmacology, and/or the biological mechanisms underpinning cocaine pathology. For instance, therapeutics that enhance cocaine metabolism (cocaine esterases) have been proposed to reverse cocaine overdose [9].

The molecular brain pathology of CUD is characterized by persistent cellular and molecular adaptations across multiple brain regions, particularly in the mesocorticolimbic “reward” pathway. Molecular adaptations in this brain pathway mediate reward, motivation, behavioral control, memory formation, incentive salience, cue, drug and stress-induced drug taking/relapse. One approach to investigate the molecular brain correlates underlying CUD (and potentially cocaine toxicity) is to examine gene expression profiles in post-mortem brain tissue by comparing individuals with CUD to matched cocaine-free controls. Currently, three studies are publicly available that assess CUD gene expression profiles in brain regions associated with addiction: the dorsal-lateral prefrontal cortex [10] (dlPFC), hippocampus [11] and midbrain [12]. Previously, medications that consistently target gene expression across meso-cortico-limbic brain regions have demonstrated robust effects for reducing binge drinking in mice [13]. Thus, screening medications that target transcriptional patterns across relevant brain regions may help identify FDA-approved therapeutics that could be repurposed for treating CUD.

Drug-discovery analyses can cut costs and time for developing effective therapeutics, which on average cost $1.4 billion and take 12–16 years to develop [14]. The NIH-funded Library of Integrated Network-based Cellular Signatures (LINCS L1000) database facilitates drug development strategies by indexing over 1.3 million expression profiles resulting from an extensive catalog of more than 48,000 perturbagens (pharmaceuticals, small molecules, shRNAs, cDNAs, and biologicals) in over 80 human cell lines [15]. This resource allows rapid screening of compounds that may target the molecular mechanisms of a disease.

**Fig. 1:**
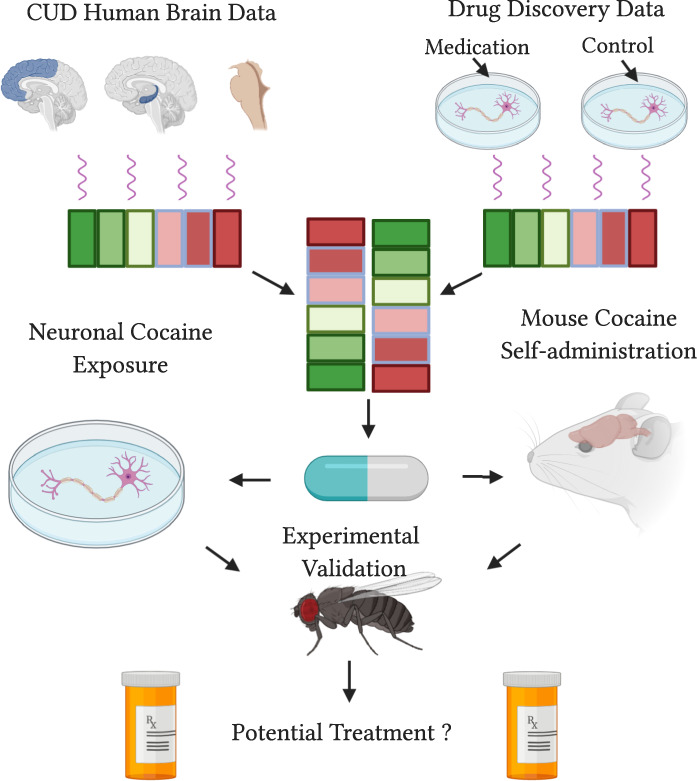
Pictoral overview of the study. This schematic outlines the stages of the study from drug discovery (top), to computational follow-up (middle) and in-vivo validation (bottom).

Here, we outline a neuronal mRNA drug-repurposing framework that identifies and validates potential medications for CUD and/or cocaine toxicity (see Fig. 1 for study overview).

## Methods

### Drug-discovery input

Our human sample (*n* = 71) used publicly available gene expression data from post-mortem brain tissue of individuals with cocaine use disorder (CUD; *n* = 36) and matched cocaine-free controls (*n* = 35). Data were obtained from three independent studies (GSM2642566 [10]; SRA029279 [11]; E-GEOD-54839 [12]; Supplementary Table S1).

We conducted drug discovery analyses leveraging the L1000 database, which catalogues mRNA expression profiles of human cell types exposed to therapeutic compounds [15]. The input for our drug discovery analysis included three categories. The first category included all differentially expressed genes (Benjamini–Hochberg False Discovery Rate (FDR) < 0.05) associated with CUD in the dlPFC [16], hippocampus [17] and midbrain [12]. Differentially expressed genes might include genes attributed to cocaine-induced toxicity, cardiac complications or psychiatric comorbidities. Thus, our second category focused on the genes related to the behavioral manifestations of CUD, by including the mRNA expression of genes from the cocaine addiction pathway (Kyoto Encyclopedia of Genes and Genomes [KEGG] database). The last category sought to further minimize potential biases of measurement error by employing “landmark” genes (those directly measured from L1000, rather than imputed) that were part of at least one of the first two categories. We refer to the input genes for our drug discovery analysis as CUD genes (Supplementary File S1).

### Drug-discovery analyses

We used a signature matching technique to find potential therapeutics for CUD or cocaine toxicity. Our analyses utilized the L1000 Connectivity Mapping gene expression profiles from the Library of Integrated Network Based Cellular Signatures (level 5 data from phase I: GSE92742 and phase II: GSE70138). Since the focus of the study was to find repurposable treatments for CUD, we selected all compounds that were FDA approved *or* in stages 1–3 of clinical trials – as listed from the drug-repurposing hub website (https://clue.io/repurposing#download-data; updated 3/24/2020). We benchmarked our findings with treatments that were currently undergoing clinical trials for CUD and specifically those that were reported on the https://clinicaltrials.gov/ website.

We focused our drug discovery analysis on two neuronal cell types used in the L1000 database: differentiated neuronal cells and neuronal progenitor cells. In total, we evaluated the potential therapeutic value of 825 compounds, which spanned 3468 individual neuronal mRNA signatures (in vitro gene expression profiles for a compound measured at a particular dose, time, and cell line). For each signature, we estimated a linear Pearson Product-Moment correlation coefficient with the CUD input. Note that not all medications undergoing clinical trials for CUD were included in the human neuronal cell lines from the L1000 database (Supplementary File S2).

To identify potential treatments for CUD, we conducted multi-level meta-regressions for all 825 compounds using the metafor package in R [18]. Multi-level meta-regression provides a powerful and interpretable framework that can accommodate complex data types. Our meta-regressions adjusted for two random effects: human brain region (or study) and the three input categories within studies (e.g. KEGG_dlPFC_, KEGG_Hippocampus_ etc.). We treated each compound as a fixed-effect incorporating the effect size (*r*) and sampling variability (*se*^2^_*r*_) for neuronal signatures of a compound. Theoretically, if the transcriptional signature of a compound is negatively associated with a disease, this compound may ‘reverse’ the underlying disease mechanisms and *may* increase the likelihood of demonstrating clinical utility. Hence, we emphasized compounds with negative meta-regression coefficients that also survived correction for multiple testing (FDR < 0.05) and defined these compounds as *potential* therapeutics. As medications with positive associations may also exert a clinical benefit, we also explored medications that were positively associated with CUD and report on these in the supplement.

We used a computational follow-up approach to prioritize the potential therapeutics identified from our drug discovery analysis using two independent transcriptome-wide datasets. We queried gene expression studies via GEO on March 2020 to find gold standard preclinical models of rodent self-administration datasets that matched brain regions and cell types relevant to the human post-mortem data. The datasets used in our study included a human neuronal cocaine exposure model [19] (SH-SY5Y neuroblastoma cells^;^ GSE71939) and a mouse model of cocaine self-administration [20] (GSE110344). The neuronal cocaine exposure dataset included 18 samples that assessed mRNA expression (via microarray) for two time points (6 or 24 h post exposure) across three doses (0*μ*Μ, 1*μ*M and 5*μ*M; the latter two mimic clinically relevant cocaine levels found in individuals with cocaine abuse). The mouse RNA-sequencing (RNA-seq) data were collected from 12–15 male C57BL/6J mice using similar brain regions to the human samples (ventral tegmental area (VTA), hippocampus and PFC). Mice pressed a lever to receive intravenous infusions of cocaine (1 mg/kg; 2 h sessions for 2 weeks) or saline. Brain tissue was extracted 24 h after the last self-administration session and mouse modeling was limited to orthologous genes listed from the mouse genome informatics dataset (http://www.informatics.jax.org/).

To maintain consistency, we harmonized the data processing and analyses for our validation datasets to resemble the human brain data (see Supplementary Methods). Using multi-level meta-regression, we assessed whether the potential CUD treatments were also negatively associated with the differential expression of the CUD genes in the neuronal cocaine exposure and mouse cocaine self-administration datasets—accounting for dose and time (neuronal exposure data) or brain region (mouse data) as random effects. Results from the computational follow-up were used to prioritize medications for experimental validation.

### Behavioral assays

*Drosophila melanogaster* (Canton-S B strain) were reared on cornmeal-molasses-yeast medium at 25 °C and 70% humidity under a 12 h light-dark cycle (lights on at 6:00 am). Cocaine-HCl was obtained from the National Institute on Drug Abuse under Drug Enforcement Administration license RA0443159. Flies were food-deprived for 24 h followed by a 20 m free-feeding period. During this period, flies consumed one of four food formulations containing either no treatment (*n* = 66 females, *n* = 61 males), cocaine only (*n* = 141 females, *n* = 140 males), cocaine and ibrutinib (*n* = 142 females, *n* = 132 males) or ibrutinib only (*n* = 68 females, *n* = 66 males). For all conditions, we observed no significant differences between ibrutinib treated flies and flies undergoing no treatment (all *t* = 0.37994, all *p* > 0.704). Thus, we reported and visualized the differences between the other three groups. The concentrations of cocaine-HCl and ibrutinib (Tocris Bioscience; Bristol, UK; Product No: 6813; Batch No: 2A/247900) were both 0.16% (w/w). The concentrations of cocaine used were based on optimal concentrations that give rise to phenotypic effects without causing lethality determined previously [21].

We quantified cocaine consumption in the *Drosophila melanogaster* model. All food formulations included 1% (w/w) FD&C Blue #1 dye. Following behavioral testing for each fly, dye was extracted into 300 µL of deionized H_2_O using Fisher brand Bead Mill 4 (Speed: 4 m/s; Time: 15 sec). To settle fly debris, tubes were centrifuged at 13,000xRCF for 1 min using an Eppendorf Centrifuge 5430R. To quantify consumption, 100 µL of each extract was dispensed in duplicate into wells of a 384-well microplate. Absorbance was measured at 630 nm using a SpectraMax iD5 Microplate Reader. Dye concentrations were calculated using a standard curve (FD&C Blue #1 in deionized H2O; 0.0–6.0 µg/mL).

Immediately following exposure, each fly underwent behavioral testing for startle response induced by a mechanical disturbance. A vial housing a single fly was dropped through a chute from a height of 42 cm and then secured in a horizontal position. During the next 45 s, the total time spent moving and the occurrence of seizures were recorded. Seizure activity was defined as significant disruption of normal movement with severe tremors and muscle twitching (see Supplementary File S3). While immobile, seizing, or grooming, flies were considered stationary. Startle response data were analyzed using a two-way fixed effects factorial ANOVA model: *Y* = *M* + *S* + *T* + S*T + *ε*, where *Y* is the phenotype, *M* is the mean, *S* is sex, *T* is treatment, *S* * *T* is the sex by treatment interaction, and *ε* is the residual. We used the type III Sums of Squares due to differing sample sizes across groups. Post-hoc comparisons were performed using Student’s *t* tests. Seizure data were analyzed using Fisher’s exact tests in R.

## Results

### Drug-discovery input

A schematic overview of the study design is presented in Fig. 1. Prior to our drug discovery analysis we examined the input data. We observed minimal overlap among differentially expressed genes identified across human brain regions (see Supplementary File S1). Associations among KEGG cocaine addiction genes were modest and sometimes negative (*r*_midbrain_hippocampus_ = −0.31, *p* = 0.044; *r*_dlPFC_hippocampus_ = 0.02, *p* = 0.915; and *r*_midbrain_dlPFC_ = 0.36, *p* = 0.019). This heterogeneity and individual variation may be one factor contributing to the difficulty in finding an effective treatment that targets multiple brain regions and is effective for all individuals in a population.

### Drug-discovery analyses

**Fig. 2:**
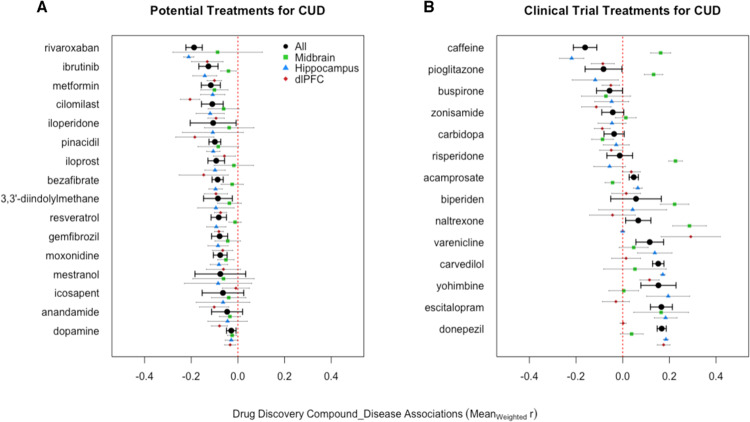
Drug-discovery results for CUD using human brain data. Panel (**A**) shows repurposable compounds negatively associated with CUD gene expression in the brain (all *p*_adj_ < 0.05). Panel (**B**) shows the results of the medications currently undergoing clinical trials for CUD. The *x*-axis represents the weighted average of a compound’s correlation coefficients with the human brain CUD genes and each compound’s weighted standard error bars. Also, note that the black circles represent the effect across all brain regions/studies and the points below are color coded by brain region.

Our drug discovery analysis discovered 16 medications with gene expression signatures that were negatively associated with gene expression associated with CUD in the midbrain, hippocampus and dlPFC (*p*_adj_ < 0.05; Fig. 2A). These medications had diverse pharmacological mechanisms of action (Supplementary Table S2) and outperformed the current pharmaceuticals undergoing clinical trials for CUD (Fig. 2B). We also show the medications that were positively associated with CUD in the supplement (see Supplementary Fig. S2; see Supplementary File S4 for all results).

**Fig. 3:**
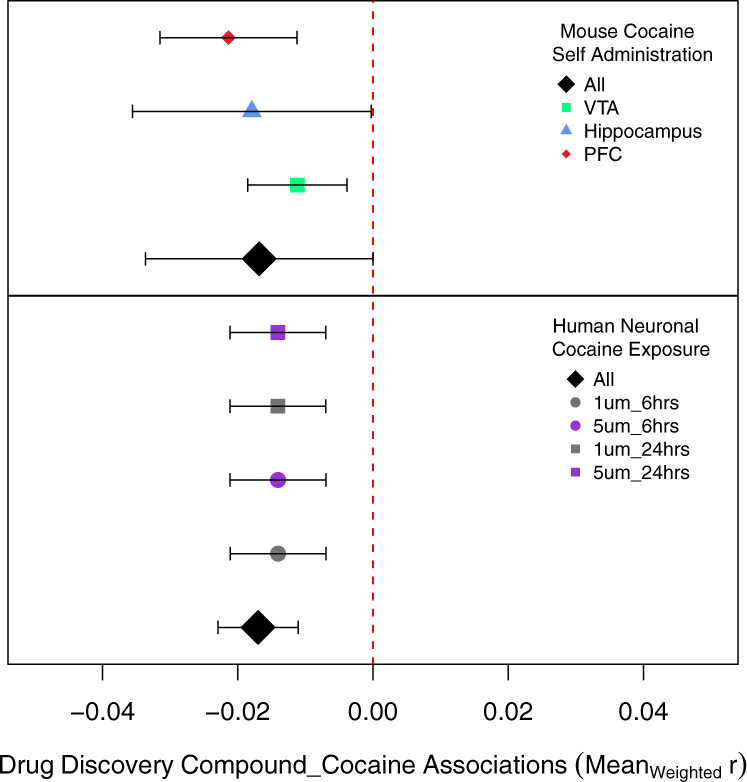
Computational follow-up in preclinical models of cocaine use. The top panel shows associations between the neuronal signatures of ibrutinib and differential expression results from the in vitro cocaine neuronal exposure data. The bottom panel displays the associations between the neuronal signatures of ibrutinib with differential expressed genes from the mouse cocaine self-administration dataset.

**Fig. 4:**
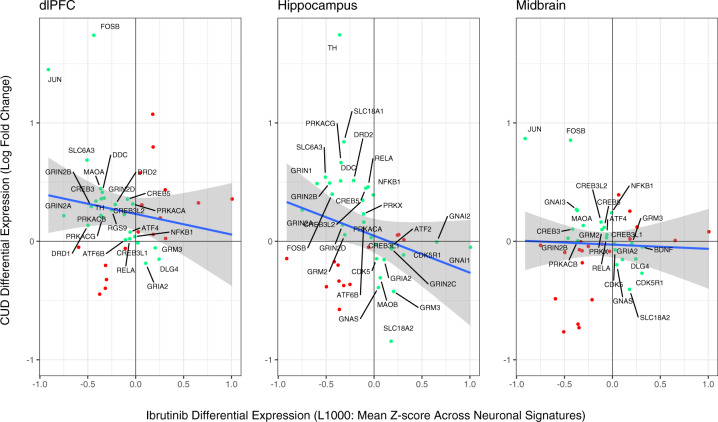
Neuronal signatures of ibrutinib are negatively associated with cocaine addiction genes in the human brain. The blue line shows the best fitting line and standard error between CUD gene expression (of KEGG Cocaine Addiction Pathway genes) and neuronal signatures of ibrutinib (collapsing across dose, time and cell line; *r*_dlPFC_ = −0.16, *r*_hippocampus_ = −0.25 and *r*_midbrain_ = −0.04). Genes were labeled if they were in the negative diagonal of the plot (green points). Differential expression of *FOSB* in the dlPFC was windsorized for visualization purposes.

Next, we employed a computational follow-up of the 16 potential CUD medications using transcriptome-wide data from preclinical in vitro and in vivo models. Only one compound, *ibrutinib*, was negatively associated with neuronal cocaine exposure (*M*_*r*_ = −0.017, se_*r*_ = 0.006, *p* = 0.001; *p*_adj_ = 0.019) and was trending to be negatively associated with the gene expression profiles associated with mouse cocaine self-administration (*M*_*r*_ = −0.017, se_*r*_ = 0.007, *p* = 0.007; *p*_adj_ = 0.0732; Fig. 3). No other medication that was negatively associated with CUD expression demonstrated significant evidence among preclinical models (see Supplementary Fig. S3). Using correlations between human CUD brain data and the in vitro drug discovery data, we found negative associations between ibrutinib and the expression of genes involved in dopaminergic and glutamatergic neurotransmission as well as various immediate early genes, intracellular signaling cascade genes and putative neuroepigenetic transcripts (Fig. 4).

### Validation of potential therapeutic efficacy of ibrutinib in the Drosophila model

To validate the effectiveness of ibrutinib as a potential therapeutic for CUD, we evaluated its effects on cocaine-induced phenotypes in the *Drosophila melanogaster* model. Drosophila provides an advantageous model system for studies on cocaine consumption [22]. The Drosophila dopamine transporter contains a binding site that can accommodate cocaine [23], and exposure to cocaine gives rise to motor responses that resemble behaviors observed in rodents. In addition, flies develop sensitization to repeated intermittent exposure to cocaine [24, 25].

**Fig. 5:**
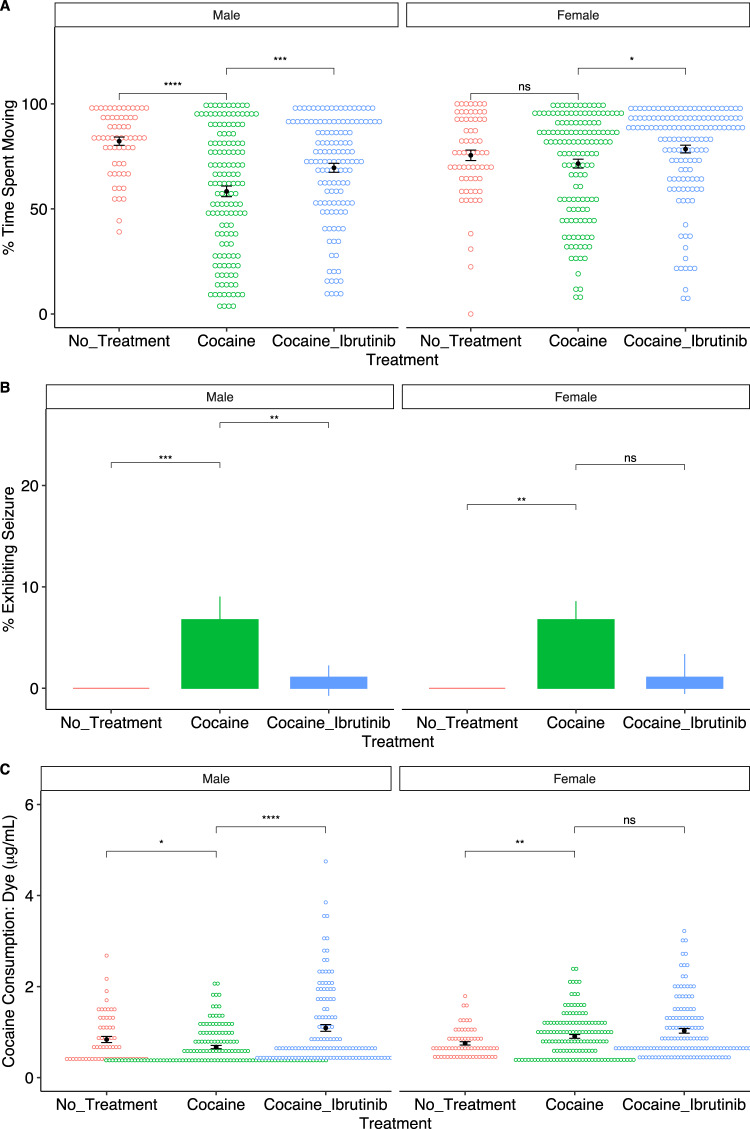
Ibrutinib influences cocaine-induced startle response, cocaine-induced seizures and cocaine consumption in *Drosophila melanogaster*. Sample sizes were as follows: *n* = 61 (♂control), 140 (♂cocaine), 132 (♂cocaine + ibrutinib), 66 (♀control), 141 (♀cocaine), 142 (♀cocaine + ibrutinib). **A** Dotplot showing the cocaine startle response by condition. **B** Barplot showing the average seizure activity during the startle response across conditions with 95% confidence intervals. **C** Dotplot showing cocaine consumption across conditions. Colored dots represent individual flies and the black dot displays the average for each group and the standard error.ns *p* > 0.05. *p < 0.05. **p < 0.01. ***p < 0.001. ****p < 0.001.

We measured cocaine consumption, startle behavior and the prevalence of seizure activity in male and female flies following acute consumption of solid food, solid food supplemented with cocaine, or solid food supplemented with cocaine and ibrutinib. Male flies exposed to cocaine spent less time moving after being subjected to a mechanical startle (Fig. 5A). This is likely due to the occurrence of cocaine-induced seizures, which were scored as stationary periods. Both male and female flies that consumed ibrutinib with cocaine spent more time moving than flies that only consumed cocaine (Fig. 5A). Male flies that consumed ibrutinib and cocaine showed a significant decrease in the prevalence of seizures (Fig. 5B). Fewer cocaine-induced seizures were also observed in Ibrutinib treated females, but this observation did not reach statistical significance since the incidence of cocaine-induced seizures was lower in females than in males. To ascertain that ibrutinib did not reduce food intake, we measured cocaine consumption with food supplemented with ibrutinib alone and found that ibrutinib increased cocaine consumption in male flies and was trending to do so in female flies (see Fig. 5C; see Supplementary File S5 for data). Altogether, these results indicate that ibrutinib may be useful as a therapeutic to prevent various neurobehavioral and toxic effects of cocaine use.

## Discussion

We identified potentially repurposable medications for CUD and/or cocaine toxicity, which demonstrated more reproducible associations across the brain’s reward circuitry than current medications undergoing clinical trials in the US. Despite the low correspondence of gene expression across datasets, ibrutinib was consistently negatively associated with patterns of gene expression observed in brains of individuals with CUD across brain regions and diverse samples as well as across two gold standard preclinical models. Ibrutinib decreased cocaine-induced seizures in the Drosophila model and thus is a promising repurposable medication for cocaine-induced seizures and possibly for other aspects of CUD that cannot readily be evaluated in Drosophila. We did not attempt to validate medications positively associated with CUD (see Supplementary Fig. S2).

Ibrutinib is an irreversible Bruton’s tyrosine kinase (BTK) inhibitor and is approved by the FDA for various B cell cancers including chronic lymphocytic leukemia [26]. BTK inhibitors have demonstrated efficacy for multiple sclerosis [27]—via an anti-inflammatory mechanism—and have also reduced binge drinking in mice [13]. BTKs are predominantly expressed in microglia in the brain (https://www.brainrnaseq.org/) and amplify intracellular signals from (B cell) receptors to the nucleus [28]. Cocaine activates overlapping intracellular signaling pathways with canonical B cell signaling pathways (e.g., *JUN, RELA,* and *NFKB1* genes) and is associated with a pro-inflammatory response in the brain [29]. We postulate that inhibiting BTK via ibrutinib interferes with the cocaine response via disruption of intracellular signaling cascades and/or evoking anti-inflammatory processes.

In addition to ibrutinib, we discovered other potential medications that may correlate with brain profiles of CUD. Some of these compounds may target molecular processes underlying chronic cocaine use. For instance, we found that dopamine was negatively associated with the differential expression of CUD genes across brain regions, which may counteract the hypodopaminergic state induced by chronic cocaine use [30]. Various drugs identified by our analyses have been previously used to treat cardiac complications, such as Rivaroxaban, Pinacidil, Iloprost, Gemfibrozil, Bexafibrate and Moxonidine. It is possible that these medications exert a protective role for the cardiovascular consequences of chronic cocaine use. Some of these medications may have both health and behavioral benefits. For instance, Moxonidine reduces (cue-induced) cocaine relapse [31] and ethanol withdrawal [32, 33] in rodents. Of note, the pharmacological profiles of the potential CUD medications we identified have shown to reduce various drug use behaviors (PPAR alpha agonists [34]; PDE4 inhibitors [35, 36]). Potential CUD medications also influenced catecholamine neurotransmission, potassium channels, hormonal signaling and mitochondrial processes, which have all been implicated in the pathophysiology of cocaine use [37–40]. Our approach may serve a dual purpose for indirectly unraveling molecular brain features of human CUD while also identifying specific chemical compounds that may demonstrate clinical utility for cocaine-related outcomes.

The results of our study should be interpreted with caution until further validation studies are conducted in different species and behavioral paradigms. We used all extant transcriptome-wide brain data on human CUD, but these sample sizes were small, ascertained from highly selected male cocaine users and generally included heterogenous cell types in the brain. These data are unable to differentiate specific symptoms of CUD and may have bias relating to health and psychiatric comorbidities. Whereas the Drosophila model can evaluate some behavioral effects following acute consumption of cocaine, many aspects of cocaine use, addiction, withdrawal, and death cannot be assessed in the fly model, but require complementary studies in other model systems. Future studies in mammalian models are warranted to determine the efficacy, dose, and safety of ibrutinib for cocaine-related outcomes. The L1000 drug discovery database assesses acute gene expression profiles of medications in human cell lines and is thus limited in understanding chronic therapeutic administration and in vivo mechanisms of medications. The lead repurposable drug from this study, ibrutinib, may not be an accessible treatment option to many, as it can be costly and while it is generally well tolerated it can have various side effects (e.g., diarrhea, infection and fatigue) [41] and off target effects (arrhythmias, platelet dysfunction, QTc prolongation) that may limit its utility for cocaine-related disease indications. Additionally, it is unknown what dose would be most effective and whether negative health consequences arise when combining this medication with cocaine (or crack cocaine). Despite these limitations, ibrutinib emerges as a promising therapeutic with potential, at least for the treatment of cocaine-induced seizures. In a broader context, our study provides a proof of principle for a powerful, flexible and interpretable strategy that can be used to identify and prioritize therapeutics from genome-wide and transcriptome-wide datasets.

## Supplementary information

Supplementary Information

Supplementary File S1

Supplementary File S2

Supplementary File S3

Supplementary File S4

Supplementary File S5
