# Supplementary material for: Ibrutinib as a potential therapeutic for cocaine use disorder
Source: Transl Psychiatry. 2021 Dec 8;11:623. doi: 10.1038/s41398-021-01737-5 (PMC8654982; doi:10.1038/s41398-021-01737-5)
Supplement: Supplementary file 1 — Supplementary Information [file 41398_2021_1737_MOESM1_ESM.docx]

**Supplementary Information**

*Supplementary Methods*

*Data Processing and Analysis*

We analyzed data from two microarray studies: human midbrain samples^1^ and *in vitro* neuronal cocaine exposure data^2^. Human midbrain samples contained three technical replicates for each individual. Correlations across technical replicates were high (all r > 0.95). Thus, we averaged across individual samples before normalization, which mitigates potential biases of batch effects. The *in vitro* neuronal cocaine exposure data only contained biological replicates, but batch effects were controlled for using surrogate variable analyses^3^. All microarray samples contained multiple array ids for individual genes. To obtain a single expression value for a gene we used the *collapseRows* function that refined all array ids into feature intensity recordings for specific genes. All genes’ feature intensity recordings were variance stabilized (via *vsn2* command^4^) and then loess normalized (via the *normalizeCyclicLoess* command^5^) before differential expression analysis. Our study used a linear empirical Bayes to obtain differential expressed genes for microarray data via the *eBayes* command^5^.

Our study also utilized three separate RNA-sequencing (RNA-seq) samples: human dorsal-lateral prefrontal cortex^6^ (dlPFC), human hippocampus^7^ and mouse self-administration data^8^ (PFC, hippocampus and ventral tegmental area (VTA)). Our RNA-seq analyses performed standard differential expression analyses using DESeq2^9^. Briefly, all human and mouse data were normalized with the *estimateSizeFactors* command that performs a standard median ratio method. Samples that were < 2 standard deviations from the mean normalization value were considered outliers and removed (one sample from the human hippocampus). Subsequently, dispersion was adjusted for via the *estimateSizeFactors* command that performs a Cox Reid-adjusted profile likelihood maximization function. Controlling for batch effects (via svaseq^10^; 2 surrogate variables), we then used the *nbinomWaldTest* command that leverages a generalized linear model and assumes a negative binomial distribution to estimate differential expression.

To be consistent with the post-mortem human brain analyses, computational follow-up using mouse and in vitro cocaine use data included differentially expressed genes associated with human CUD as well as the genes from the Cocaine Addiction KEGG pathway.

| Post-Mortem Human Sample Information: M (s.d.) [n] | | | |
| --- | --- | --- | --- |
| Attribute | dlPFC [n = 36] | Hippocampus [n = 15] | Midbrain [n = 20] |
| Specific Brain | Broadmann's Area 46 | CA4-CA1 | VTA & SN |
| Region |  | Dentate gyrus |  |
| CUD | DSM-V | DSM-IV Abuse | DSM-IV Abuse |
| Criteria |  | or Dependence |  |
| Technology | RNA-seq | RNA-seq | Microarray |
| n [case / control] | [19 / 17] | [7 / 8] | [10 / 10] |
| Sex | 100% Male | 100% Male | 100% Male |
| Age | 35.0 (11.0) | 39.4 (6.4) | 49.2 (3.9) |
| Ethnicity | European American or Hispanic [23] | European American or Hispanic [10] | European American or Hispanic [4] |
|  | African American [13] | African American [5] | African American [16] |
| PMI | 16.5 (6.4) | 16.9 (4.2) | N/A |
| pH | 6.4 (0.3) | N/A | 6.5 (0.2) |
| RNA Integrity | 2.9 (0.8) | N/A | 6.5 (0.9) |
| Blood: Cocaine (mg/L) | 6.1 (10.8) | 3.3 (4.9) | 0.3 (0.3) |
| Brain: Cocaine (mg/L) | 5.9 (8.4) | 1.6 (1.4) | N/A |
| Blood: BE (mg/L) | 4.6 (4.6) | 3.6 (4.1) | N/A |
| Brain: BE (mg/L) | 1.5 (2.0) | 1.1 (1.8) | N/A |

**Supplementary Table S1:** Descriptive Statistics on Brain Tissues and Samples

Note the incomplete information across samples (e.g., missing PMI [post-mortem interval], RNA integrity, incomplete information on ethnicity, BE [benzoylecgonine] and other aspects).

**Supplementary Figure S1** Negligible Overlap of Differentially Expressed Genes Associated with CUD (FDR < 0.05) Across Brain Regions / Study


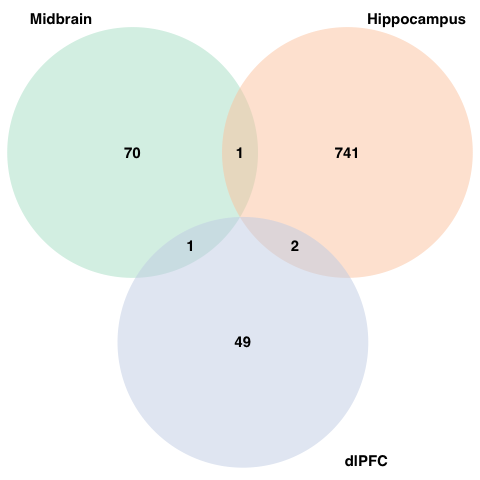


Note these are the # of genes per each brain region that were also included in the L1000 drug discovery dataset.

**Supplementary Table S2** Descriptive Information on the Potential Therapeutics Identified for CUD

| **Proposed Mechanisms and Gene Targets for the Potential Repurposable Medications for CUD or Cocaine Toxicity** | | |
| --- | --- | --- |
| Compound | Mechanism of Action | Gene Targets |
| **ibrutinib** | Bruton's tyrosine kinase (BTK) inhibitor | *BLK, BMX, BTK* |
| rivaroxaban | coagulation factor inhibitor | *F10* |
| pinacidil | ATP channel activator & | *ABCC8, ABCC9* |
|  | potassium channel activator |  |
| iloperidone | dopamine receptor antagonist & | *ADRA1A, ADRA2C, DRD1, DRD2, DRD3, DRD4,* |
|  | serotonin receptor antagonist | *HRH1, HTR1A, HTR2A, HTR6, HTR7* |
| metformin | insulin sensitizer | *ACACB, PRKAB1* |
| anadamide | cannabinoid receptor agonist | *CACNA1G, CACNA1H, CACNA1I, CNR1, CNR2* |
|  |  | *GLRA1, GPR18, GPR55, KCNA2, KCNK3, KCNK9, TRPM8, TRPV1* |
| icosapent | platelet aggregation inhibitor | *ACSL3, ACSL4, FADS1, FFAR1, PPARD,* |
|  |  | *PPARG, PTGS1, PTGS2, SLC8A1, TRPV1* |
| dopamine | dopamine receptor agonist | *DBH, DRD1, DRD2, DRD3, DRD4, DRD5,* |
|  |  | *HTR1A, HTR7, SLC6A2, SLC6A3, SLC6A4* |
| iloprost | platelet aggregation inhibitor & | *PTGDR, PTGER1, PTGER2, PTGER3,* |
|  | prostanoid receptor agonist | *PTGER4, PTGFR, PTGIR, TBXA2R* |
| cilomilast | phosphodiesterase inhibitor | *PDE4A, PDE4B, PDE4D* |
| reservatrol | cytochrome P450 inhibitor, SIRT activator | *CSNK2A1, NQO2, PTGS1, PTGS2* |
| mestranol | estrogen receptor agonist | *ESR1* |
| gemfibrozil | lipoprotein lipase activator | *LPL, PPARA, SLCO1B1, SLCO1B3, SLCO2B1* |
| bezafibrate | PPAR receptor agonist | *PPARA, PPARD, PPARG* |
| moxonidine | imidazoline receptor agonist | *ADRA2A, ADRA2B, ADRA2C* |
| 3,3' - diindolylmethane | CHK inhibitor, cytochrome P450 activator & | *AR, HIF1A, IFNG, PI3* |
|  | indoleamine 2,3-dioxygenase inhibitor |  |
| rivaroxaban | coagulation factor inhibitor | *F10* |

**Supplementary Figure S2** Repurposable medications for CUD

**
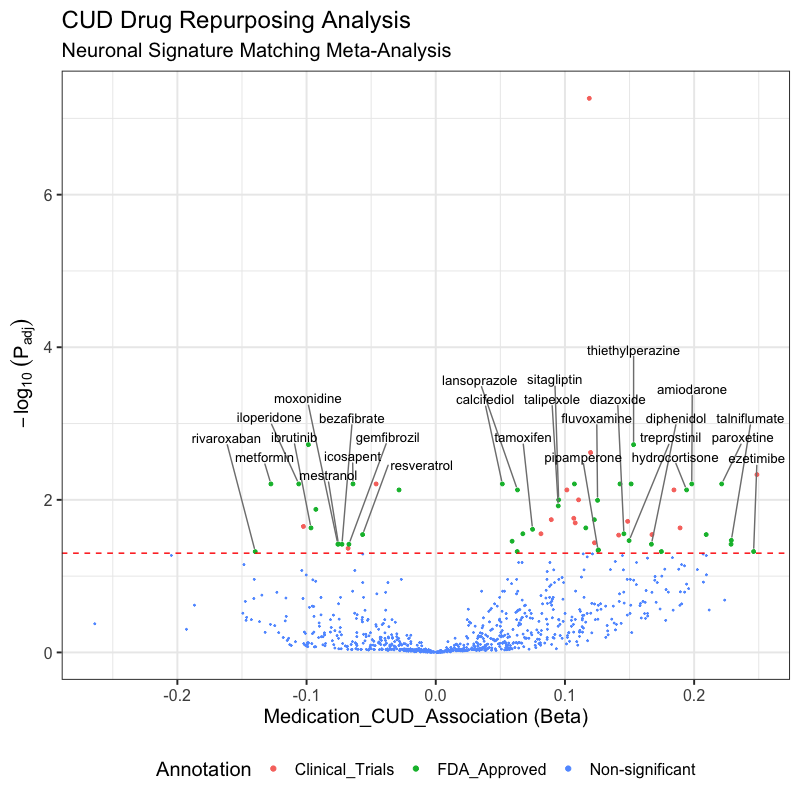
**

Volcano plot showing results from drug discovery analysis of human CUD. X-axis shows the association of a medication with CUD and the y-axis indicates the significance of a medication. The dashed red line denotes the threshold for significance (FDR < 0.05). Medications with negative values would be negatively associated with the CUD signatures in brain tissue across studies. Labeled medications are FDA approved, in pill form and cross the blood brain barrier (as identified by DrugBank: https://go.drugbank.com/).

**Supplementary Figure S3** Computational Follow-up of Potential Therapeutics for CUD in Preclinical Models of Cocaine Use


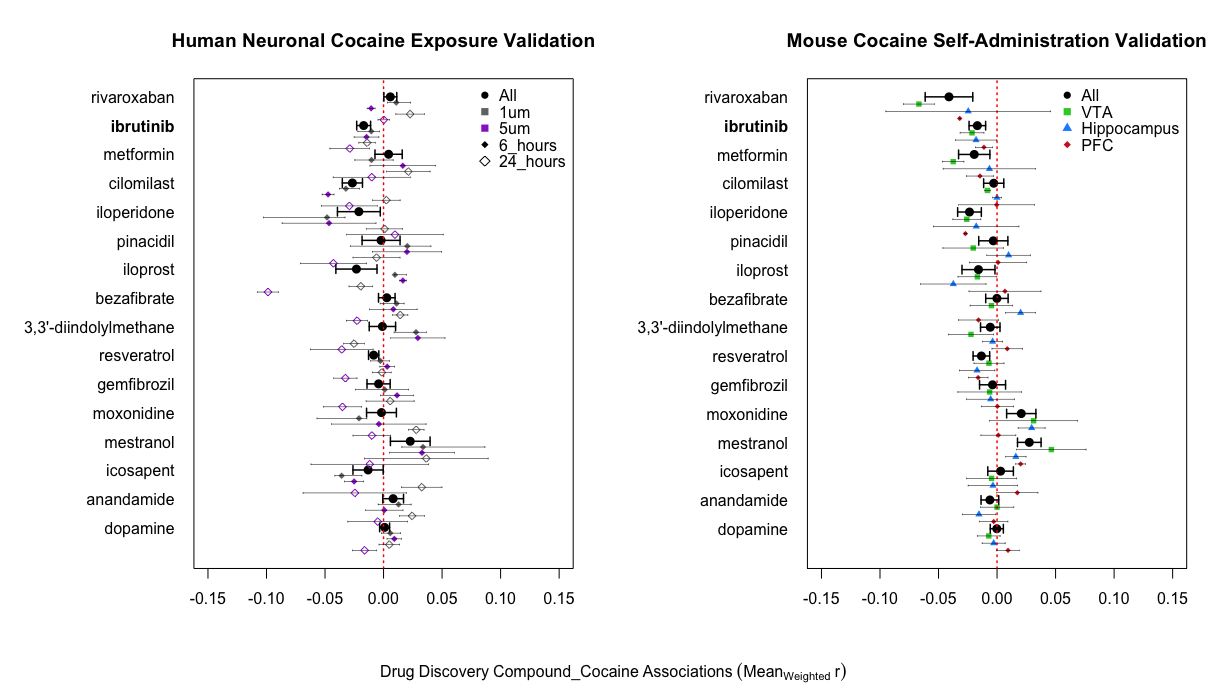


The left panel shows the results from the *in vitro* cocaine exposure dataset^2^ whereas the right panel displays findings from the *in vivo* dataset^8^. *Note* the points on the graph are color coded by dose and time (left panel) and brain region (right panel), with the black circle representing the overall effect for a compound.

**References**

1. Bannon MJ, Johnson MM, Michelhaugh SK, Hartley ZJ, Halter SD, David JA, et al. A molecular profile of cocaine abuse includes the differential expression of genes that regulate transcription, chromatin, and dopamine cell phenotype. *Neuropsychopharmacology* 2014;39(9):2191–9.

2. Fernandez-Castillo N, Cabana-Dominguez J, Soriano J, Sanchez-Mora C, Roncero C, Grau-Lopez L, et al. Transcriptomic and genetic studies identify NFAT5 as a candidate gene for cocaine dependence. *Transl Psychiatry* 2015;5:1–9.

3. Leek JT, Storey JD. Capturing heterogeneity in gene expression studies by surrogate variable analysis. *PLoS Genet* 2007 Sep 28;3(9):e161.

4. Huber W, Von Heydebreck A, Sültmann H, Poustka A, Vingron M. Variance stabilization applied to microarray data calibration and to the quantification of differential expression. *Bioinformatics* 2002 Jul 1;18(suppl_1):S96-104.

5. Ritchie ME, Phipson B, Wu DI, Hu Y, Law CW, Shi W, Smyth GK. limma powers differential expression analyses for RNA-sequencing and microarray studies. *Nucleic acids research* 2015 Apr 20;43(7):e47-.

6. Ribeiro EA, Scarpa JR, Garamszegi SP, Kasarskis A, Mash DC, Nestler EJ. Gene Network Dysregulation in Dorsolateral Prefrontal Cortex Neurons of Humans with Cocaine Use Disorder. *Sci Rep* 2017;7(1):1–10.

7. Zhou Z, Yuan Q, Mash DC, Goldman D. Substance-specific and shared transcription and epigenetic changes in the human hippocampus chronically exposed to cocaine and alcohol. *PNAS* 2011;108(16).

8. Walker DM, Cates HM, Loh YE, Purushothaman I, Ramakrishnan A, Cahill KM, et al. Cocaine self-administration alters transcriptome-wide responses in the brain’s reward circuitry. *Biol Psychiatry* 2018;84(12):867–80.

9. Love MI, Huber W, Anders S. Moderated estimation of fold change and dispersion for RNA-seq data with DESeq2. *Genome biology* 2014 Dec;15(12):1-21.

10. Leek JT. Svaseq: removing batch effects and other unwanted noise from sequencing data. *Nucleic acids research* 2014 Dec 1;42(21):e161-.
